# The complete chloroplast genome sequence of *Actinidia arguta* using the PacBio RS II platform

**DOI:** 10.1371/journal.pone.0197393

**Published:** 2018-05-24

**Authors:** Miaomiao Lin, Xiujuan Qi, Jinyong Chen, Leiming Sun, Yunpeng Zhong, Jinbao Fang, Chungen Hu

**Affiliations:** 1 Zhengzhou Fruit Research Institute, Chinese Academy of Agriculture Sciences, Zhengzhou, China; 2 Key Laboratory of Horticultural Plant Biology (Ministry of Education), College of Horticulture and Forestry Science, Hua Zhong Agricultural University, Wuhan, China; Montana State University Bozeman, UNITED STATES

## Abstract

*Actinidia arguta* is the most basal species in a phylogenetically and economically important genus in the family Actinidiaceae. To better understand the molecular basis of the *Actinidia arguta* chloroplast (cp), we sequenced the complete cp genome from *A*. *arguta* using Illumina and PacBio RS II sequencing technologies. The cp genome from *A*. *arguta* was 157,611 bp in length and composed of a pair of 24,232 bp inverted repeats (IRs) separated by a 20,463 bp small single copy region (SSC) and an 88,684 bp large single copy region (LSC). Overall, the cp genome contained 113 unique genes. The cp genomes from *A*. *arguta* and three other *Actinidia* species from GenBank were subjected to a comparative analysis. Indel mutation events and high frequencies of base substitution were identified, and the *accD* and *ycf2* genes showed a high degree of variation within *Actinidia*. Forty-seven simple sequence repeats (SSRs) and 155 repetitive structures were identified, further demonstrating the rapid evolution in *Actinidia*. The cp genome analysis and the identification of variable loci provide vital information for understanding the evolution and function of the chloroplast and for characterizing *Actinidia* population genetics.

## Introduction

The chloroplast (cp) performs photosynthesis and carbon fixation, which is a key plant cell organelle[1]. Typically, cp genomes in angiosperms are highly conserved and have a circular structure ranging from 115 to 165 kb in length and consisting of a small single copy region (SSC), a large single copy region (LSC), and a pair of inverted repeats (IRs)[2]. IRs influence the length of various cp genomes[3]. In cp genome evolution, gene losses and/or additions or transfer often occur, accompanied by speciation over time[1,4,5]. The gene contents and organization are conserved; thus, cp sequences can be used to answer evolutionary, phylogenetic and taxonomic questions. Cp genome information also provides a basis for studying photosynthesis regulation and plant resistance[6,7].

The genus *Actinidia*, which is a sister clade to *Clematoclethra* and contains 54 species and 21 varieties, is an important germplasm in the Actinidiaceae family of asterids. Analyzing the cp genome sequences will not only enhance our understanding of cp genome evolution in the kiwifruit family but also assist in resolving the phylogenetic relationships in *Actinidia*[8]. Despite the known wide diversity in the *Actinidia* genus, only a few of *Actinidia* cp genomes have been studied. Three *Actinidia* species were previously sequenced using the Illumina platform[8,9]. Among the *Actinidia* species, *Actinidia arguta* is the most widespread[10–12]. Liu[13] sequenced and analyzed the whole genome and verified that *A*. *arguta* was at the basal position in the *Actinidia* genus; however, a detailed analysis of the cp genome in *A*. *arguta* is lacking. *A*. *arguta* is an older species and has several specific characteristics, such as exhibiting earlier leaf drop in the autumn, which maybe related to cold resistance[14]. The chloroplast is a fundamental organelle for photosynthesis, and metabolism is associated with cp genome function; these characteristics increase the importance of studying the cp genome in this group. The angiosperm chloroplast genome has a uniparental inheritance and conservative structure[15]. For example, *Actinidia* cps are paternally inherited, which means that several phenotypes come only from the father, which gives us a different view when studying *Actinidia* phylogeny and character inheritance[16].

The third-generation sequencing platform PacBio RS II utilizes a single-molecule real-time (SMRT) sequencing technology, it has been successfully applied in many plant cp genome sequencing[1,17]. The main advantage of this method is the long read length of over 10 kb on average, with some reads possibly reaching up to 60 kb, these long read lengths provide many benefits in genome assembly, including longer contigs and fewer unresolved gaps[17][18]. However, PacBio technology has high rates of random error in its single-pass reads; therefore, the combination with Illumina sequencing technology can reduce these random errors.

In this study, we sequenced and analyzed the complete cp genome from *A*. *arguta* using Illumina and PacBio RS II sequencing technologies. We compared the gene contents and organization with those from other cp genomes in *Actinidia* and other angiosperms to identify useful variable loci and to determine phylogenetic relationships. Studying the *A*. *arguta* cp genome will allow us to learn more about its phylogenetic information and assist with future hybridization breeding studies.

## Results and discussion

### Genome sequencing and assembly

**Fig 1:**
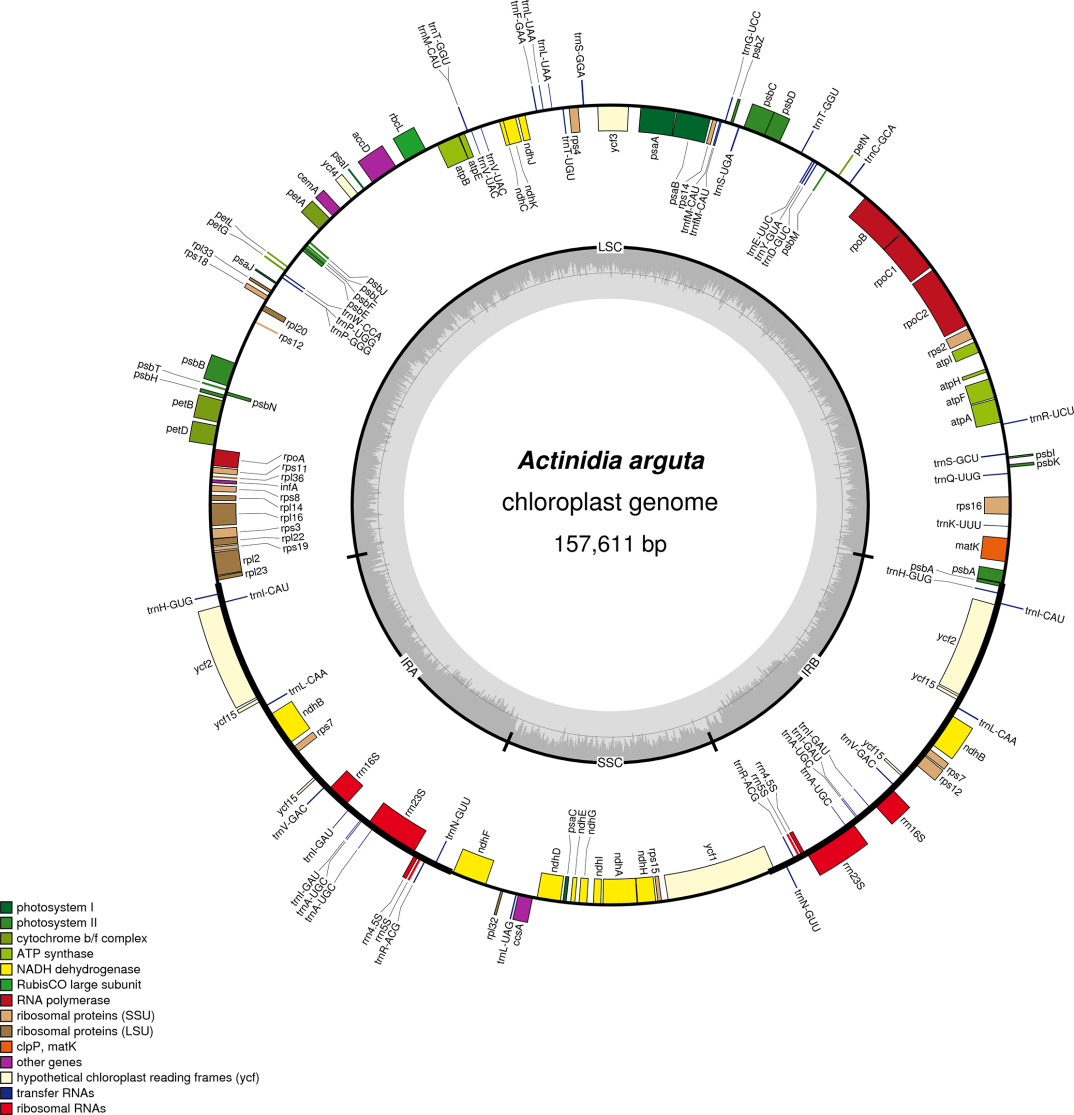
*A*. *arguta* (Actinidiaceae) genome map. Genes shown outside the outer circle are transcribed clockwise, while those inside are transcribed counterclockwise. Genes belonging to different functional groups are color coded. The dashed area in the inner circle indicates the GC content of the chloroplast genome.

Using the PacBio RS II System, 3.6 G of raw sequence data and a total of 579,226 reads were generated from *A*. *arguta*, the mean read length is 6,221 bp and the N50 contig size is 7,613 bp. The sequencing data after filtering accorded with the quality control standards. Using Illumina sequencing, 2.1 G of Illumina data and a total of 4,247,317 reads were generated. The complete cp genome for *A*. *arguta* was assembled using the PacBio RS II System. The assembled cp genome was adjusted to match the Illumina sequencing data, and one gap was filled using PCR amplification. Finally, a 157,611 bp contig with a 121× depth of coverage was assembled. The complete cp genome exhibited the typical quadripartite structure of angiosperms, consisting of a pair of inverted repeat regions (IRa and IRb, 24,232 bp) separated by an LSC (88,684 bp) and an SSC (20,463 bp) (Fig 1). The GC content of the LSC, SSC, and IRa/b regions was 35.5%, 31.05% and 42.74%, respectively, and the average GC content was approximately 37.15%. A total of 113 genes were found in the *A*. *arguta* cp genome, including 79 protein-coding genes, 4 rRNA genes, 30 tRNA genes, and 12 genes with introns. The raw data of Illumina and PacBio were deposited in SRA (SRP138693). The *A*. *arguta* cp genome sequence was submitted in GenBank (accession number: MG744576).

A similar pattern of genes of *A*. *chinensis* was also reported in previous reports[8,9], indicating the highly syntenic nature of *Actinidia* cp genomes. *A*. *arguta* was the first assembled cp genome based on the PacBio platform, which provided the most intact cp genome. Because PacBio technology sequences can avoid deletion and insertion caused by other sequencing methods, its long reads and lack bias in the coverage of AT-rich regions is preferable for highly accurate cp genomes[19].

### Divergence hotspot regions

**Fig 2:**
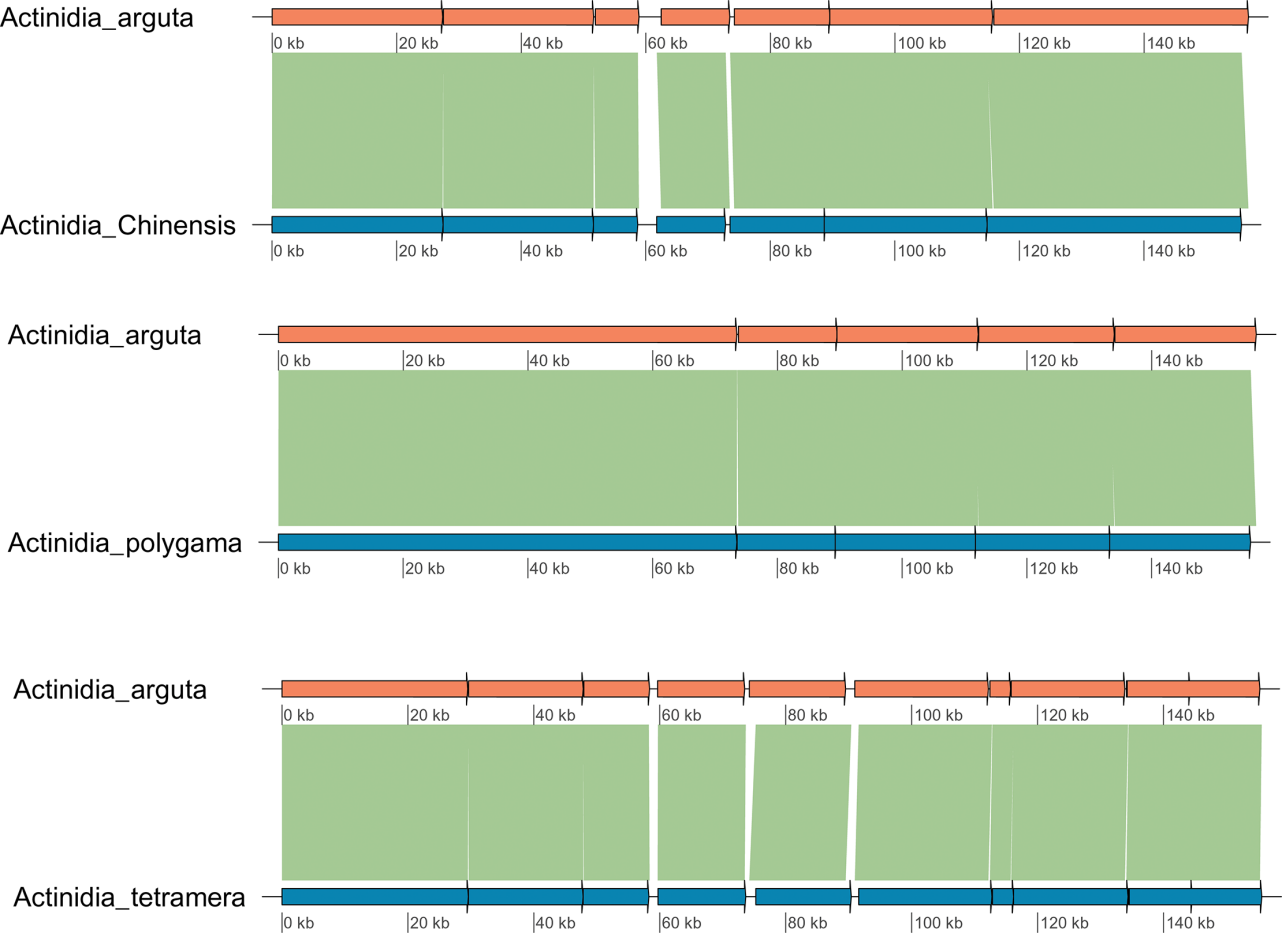
Collinearity analysis in *A*. *arguta* and three other *Actinidia* species.

**Fig 3:**
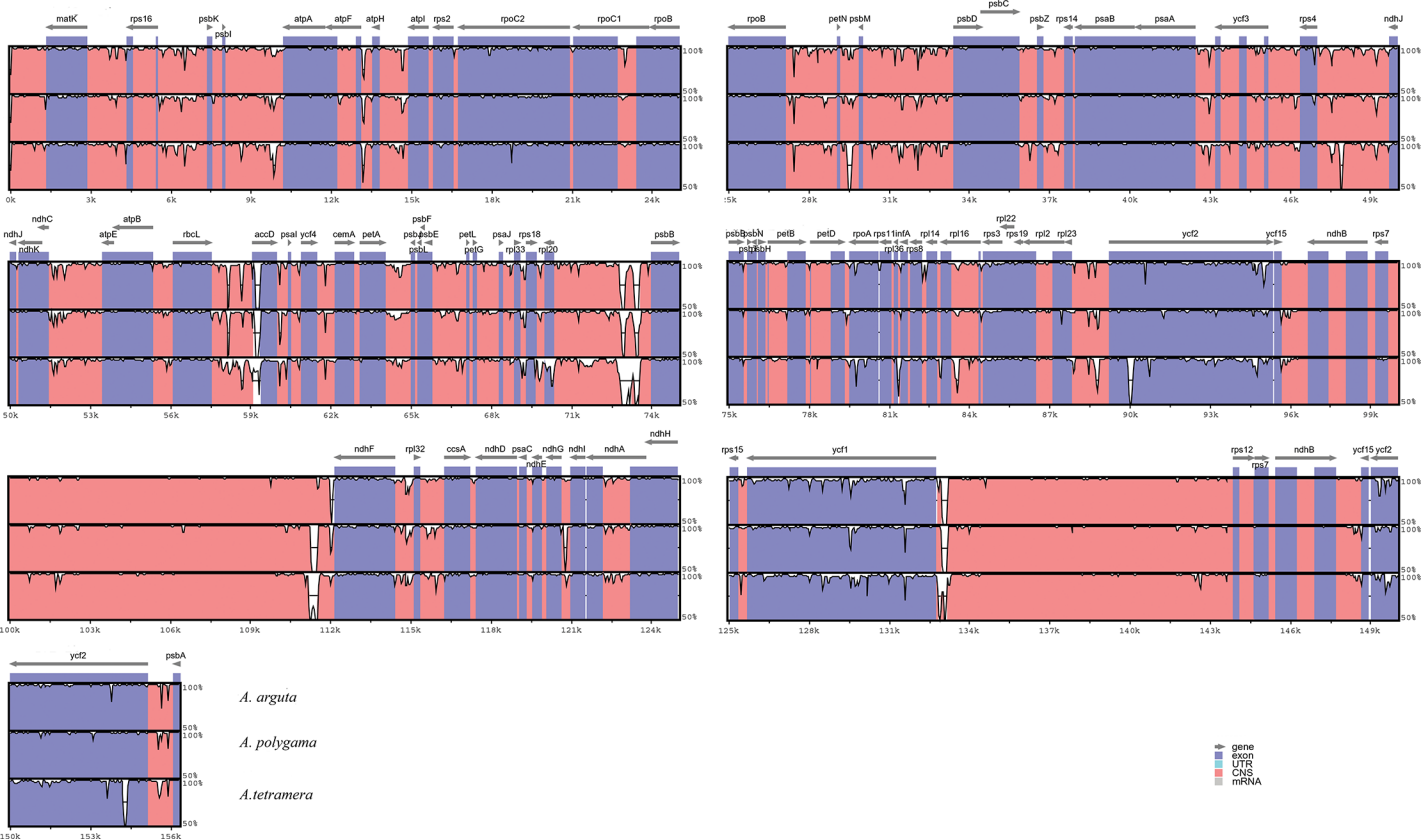
Visualization of the alignment of three chloroplast genome sequences. *A*. *chinensis* was used as the reference sequence. The vertical scale indicates the identity percentage, which ranges from 50 to 100%. The horizontal axis indicates the coordinates within the chloroplast genome. Annotated genes are displayed along the top.

The collinearity analysis revealed that the identity between the *A*. *arguta* cp genome and those from the other three *Actinidia* was ≥96%, showing a high degree of collinearity (Fig 2). The results of the divergence analysis are shown in Fig 3. The organization of the cp genome is conserved within *Actinidia*; the sequence divergence of IRa is less than that of LSC and SSC[20]. The highly divergent regions included the gene-coding regions for *accD*, *rpl20*, *ycf1*, and *ycf2*; in addition, compared with the coding regions, the noncoding regions showed higher variation, which is in agreement with similar results reported previously[21]. These divergent hotspot regions giving us abundant information for developing molecular markers for plant identification of *Actinidia* species and phylogenetic analysis.

### Repeat sequence analysis

**Fig 4:**
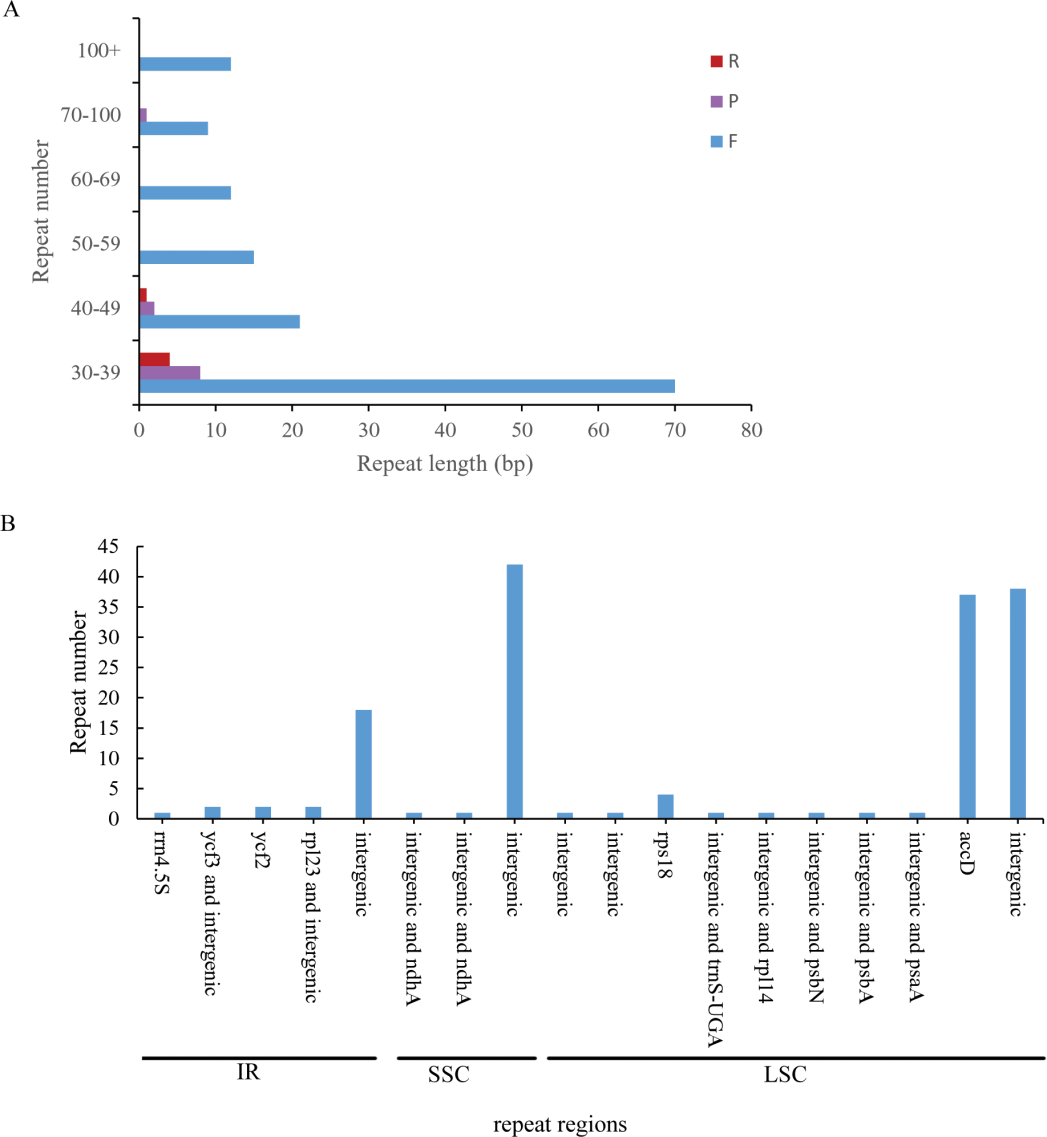
Analysis of repeat sequences in the *A*. *arguta* chloroplast genome. (A) Numbers of different repeat types detected in *A*. *arguta*. (B) Distribution of repeat sequences in the chloroplast genome.

There are a large amount of repeated sequences in cp genomes, especially in the intergenic spacer regions, have been reported in a number of angiosperm lineages, including other asterids[22]. A total of 155 repeat pairs (≥30 bp) were identified, with the following three repeat types: (1) 139 pairs were forward matches (F), (2) 11 pairs were palindromic matches (P), and (3) 5pairs were reverse matches (R). Few repeats occurred in the IR regions (25), and the repeats in the LSC and SSC numbered 86 and 44, respectively (Fig 4A). Approximately 39% of these repeats were distributed in protein-coding regions. The *rps18*, *ycf2*, *rrn4*.*5S*, *psaB*, and *accD* genes contained repeat regions (Fig 3B). In particular, the *accD* gene contained 37 repeats. Eight repeats occurred between intergenic regions and protein-coding genes, including *rps12* and intergenic, *rpl23* and intergenic, intergenic and *ndhA*, intergenic and *psbA*, intergenic and *psbN*, intergenic and *trnS*-*UGA*, intergenic and *rpl14*, and intergenic and *psaA* (S2 Table).

Repetitive structures in cps play an important role in phylogenetic and population genetics studies, as slipped strand mispairing and improper recombination of the repeat sequences results in cp genome rearrangement and variation[23]. In our results, repeats were least common in the IR regions, and the number of repeats in *A*. *arguta* overall was higher than those previously reported in other *Actinidia* species[9]. *A*. *arguta* had the fewest palindromic matches and the most forward matches. The repeats ranged from 30 to 327 bp in length, which is longer than those in *A*. *tetramera* and *A*. *polygama*[8]. These differences provide more information for elucidating *Actinidia* evolution.

### SSR analysis of the *A*. *arguta* cp genome

**Fig 5:**
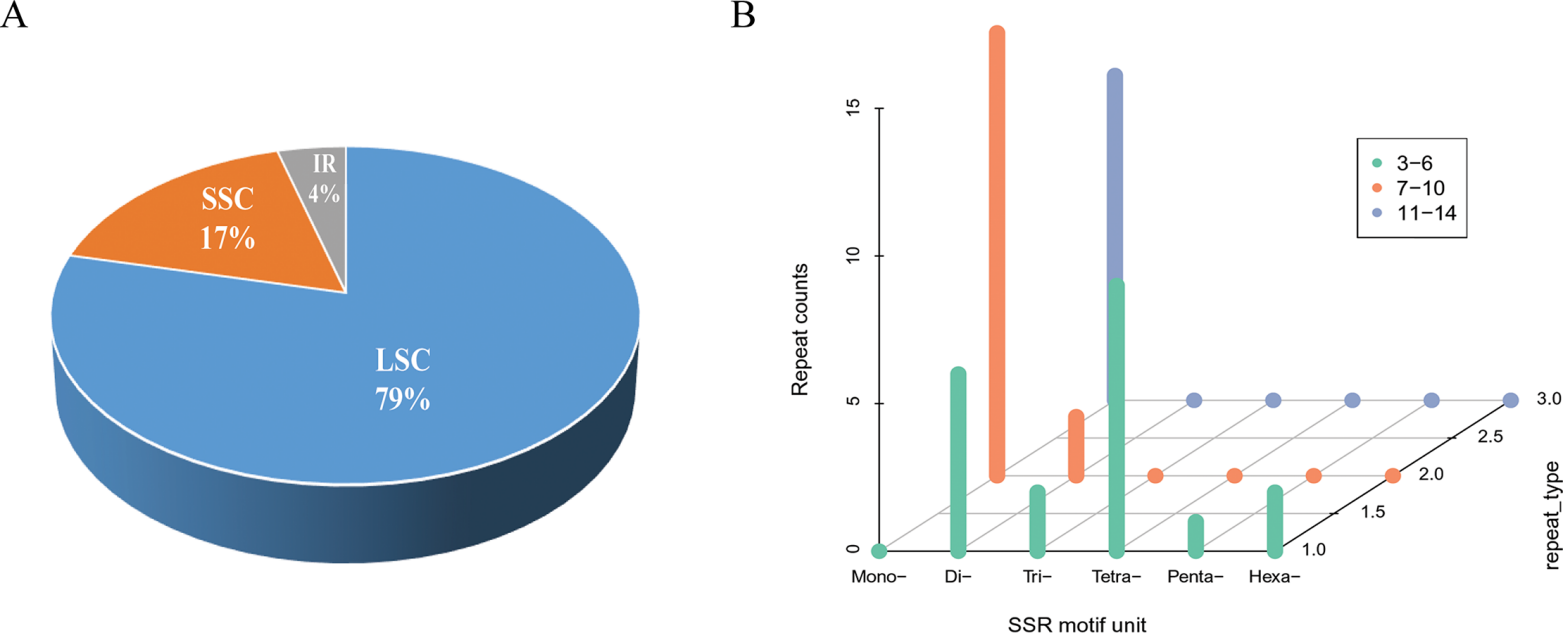
Analysis of simple sequence repeats (SSRs) in the *A*. *arguta* chloroplast genome. (A) Presence of SSRs in the LSC, SSC, and IR regions. (B) Frequency of identified SSR motifs of different repeat types.

Due to their high rates of variation in plants, simple sequence repeats (SSRs) have been used as molecular markers for understanding cp evolutionary and population genetics[24,25]. In this study, MISA analysis revealed forty-seven SSRs with a length of at least 10 bp in the *A*. *arguta* cp genome, including thirty-seven SSRs in the LSC, eight SSRs in the SSC, one in the IRa and one in the IRb. Thirty-nine of the forty-seven SSR loci are located in intergenic regions and eight are in gene coding regions (Fig 5A) (Table 1). All of the dinucleotide SSRs are composed of multiple copies of AT/TA repeats. All mononucleotides are composed of A/T and only four tetranucleotides and one pentanucleotide contain C. The *A*. *arguta* SSRs are rich in A and T, which is similar to previous reports in other species[26,27]. A greater variety of smaller SSRs were detected in *A*. *arguta* than in other *Actinidia* species. Six SSR types (mono-, di-, tri-, tetra-, penta-, and hexanucleotide repeats) were detected (Fig 5B), though the majority of the SSRs in the cp genome are mononucleotides. Interestingly, tetranucleotide repeats are the second most common SSR type in *A*. *arguta*, while the pentanucleotide type is present only in *A*. *arguta*.

**Table 1:** SSR sequences in the *Actinidia arguta* chloroplast genome. The SSR-containing coding regions are indicated in parentheses.

SSR type	Region	Number of SSRs	Start positions
(A)14	SSC	1	115,715
(AATA)3	SSC	1	118,712 (*ndhD*)
(AATC)3	SSC	1	124,042 (*ndhA*)
(T)10	SSC	10	3,060; 8,569; 26,353 (*rpoB*); 27,526; 51,891; 61,025; 82,945; 85,138; 86,675; 118,408
(T)14	SSC	1	129,212
(TAAT)3	SSC	1	115,877
(TTA)6	SSC	1	129,551
(A)10	LSC	5	3,708; 8,217; 14,745; 45,251; 77,322
(A)11	LSC	3	42,954; 44,999 (*ycf3*); 58,342
(AAAT)3	LSC	1	62,183
(AAT)4	LSC	1	6,986
(AT)5	LSC	2	20,004 (*rpoC2*); 21,037 (*rpoC1*)
(AT)6	LSC	1	55,603
(AT)7	LSC	1	36,947
(CAAT)3	LSC	1	28,092
(T)11	LSC	4	14,611; 18,661 (*rpoC2*); 31,953; 51,021 (*ndhK*)
(T)13	LSC	2	60,328; 82,443
(TA)5	LSC	2	116,718; 13,134
(TA)7	LSC	1	50,224
(TATT)3	LSC	1	48,394
(TCTT)3	LSC	1	32,812
(TTTC)3	LSC	1	66,645
(TTTC)3	LSC	1	80,223
(TTTTA)3	LSC	1	6,357
(TCTTCA)4	IRb	1	150,582
(ATGAAG)4	IRa	1	95,690

The distribution of SSRs in angiosperm cp genomes is uneven[26]. In *Actinidia*, mono-, di-, and hexanucleotide SSRs were detected in *A*. *tetramera* and mono-, di-, and trinucleotide SSRs were detected in *A*. *polygama* and *A*. *chinensis*[8,9]. In contrast, the related genus *Clematoclethra* contained only mono- and dinucleotide SSRs. In our results, *A*. *arguta* had a higher abundance of different SSR types. Therefore, the different types of SSRs can be used as cp markers because they each show unique features.

#### Variation analysis

**Fig 6:**
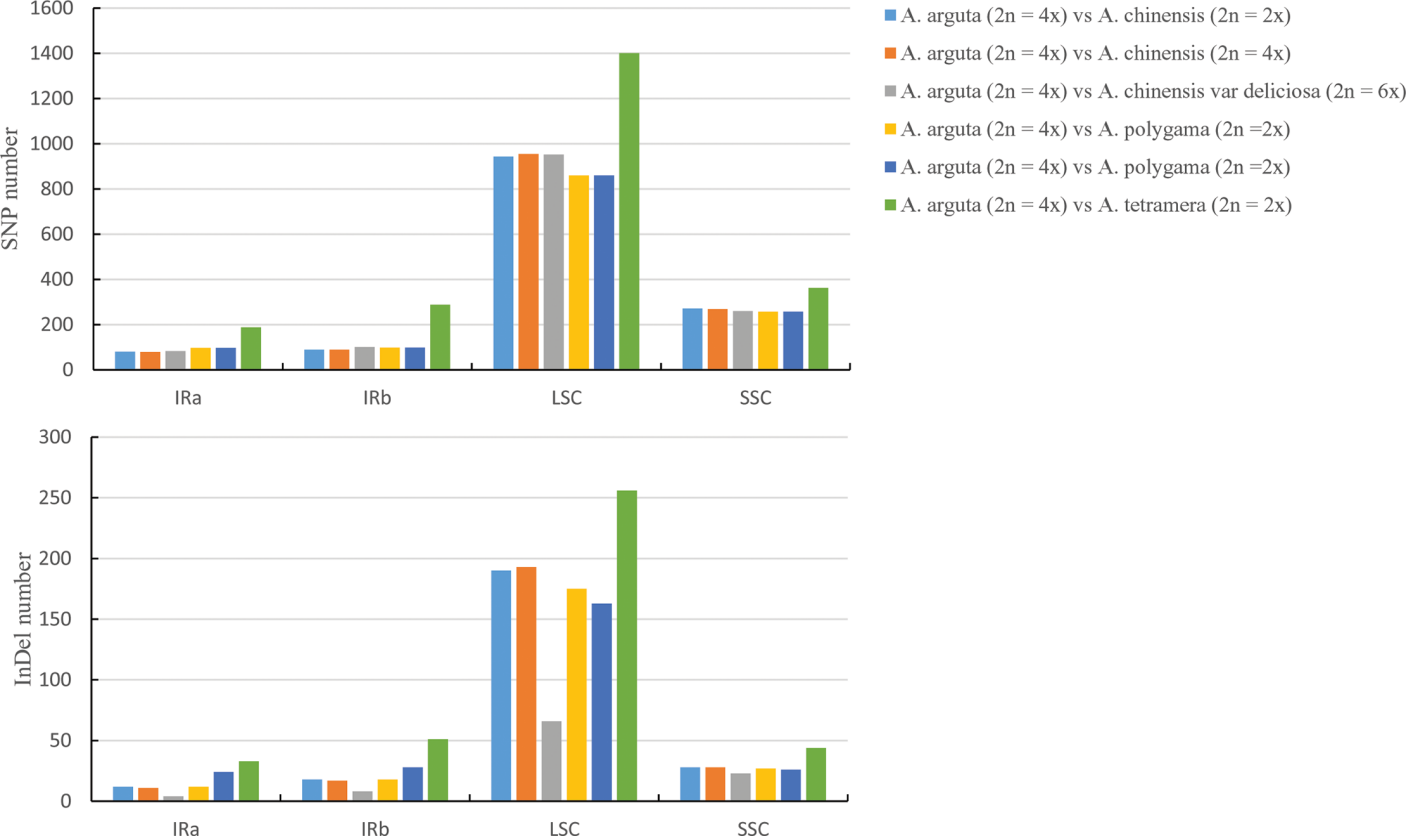
Presence of SNPs and indels between *A*. *arguta* and other *Actinidia* species.

Variations including SNPs and indels have been used as DNA markers in phylogenetic analyses for many plants[28,29]. By comparing the cp genome sequences from *A*. *arguta* with those from other *Actinidia* species, a total of 248, 249, 247, 232, 241, and 384 indels and 1386, 1392, 1429, 1396, 1313, and 2241 SNPs were identified in *A*. *chinensis* (2n = 2x), *A*. *chinensis* (2n = 4x), *A*. *chinensis* var. *deliciosa* (2n = 4x), *A*. *chinensis* var. *deliciosa* (2n = 6x), *A*. *polygama*, and *A*. *tetramera*, respectively (S3 and S4 Tables). The SNP and indel distribution in IRa, IRb, LSC, and SSC showed similar proportions. The IR regions had fewer SNPs and indels than LSC and SSC (Fig 6), which was also reported in other plants[30]. These variations in *Actinidia* can be used for developing genetic markers for screening hybrid offspring in different species.

### IR contraction and expansion

**Fig 7:**
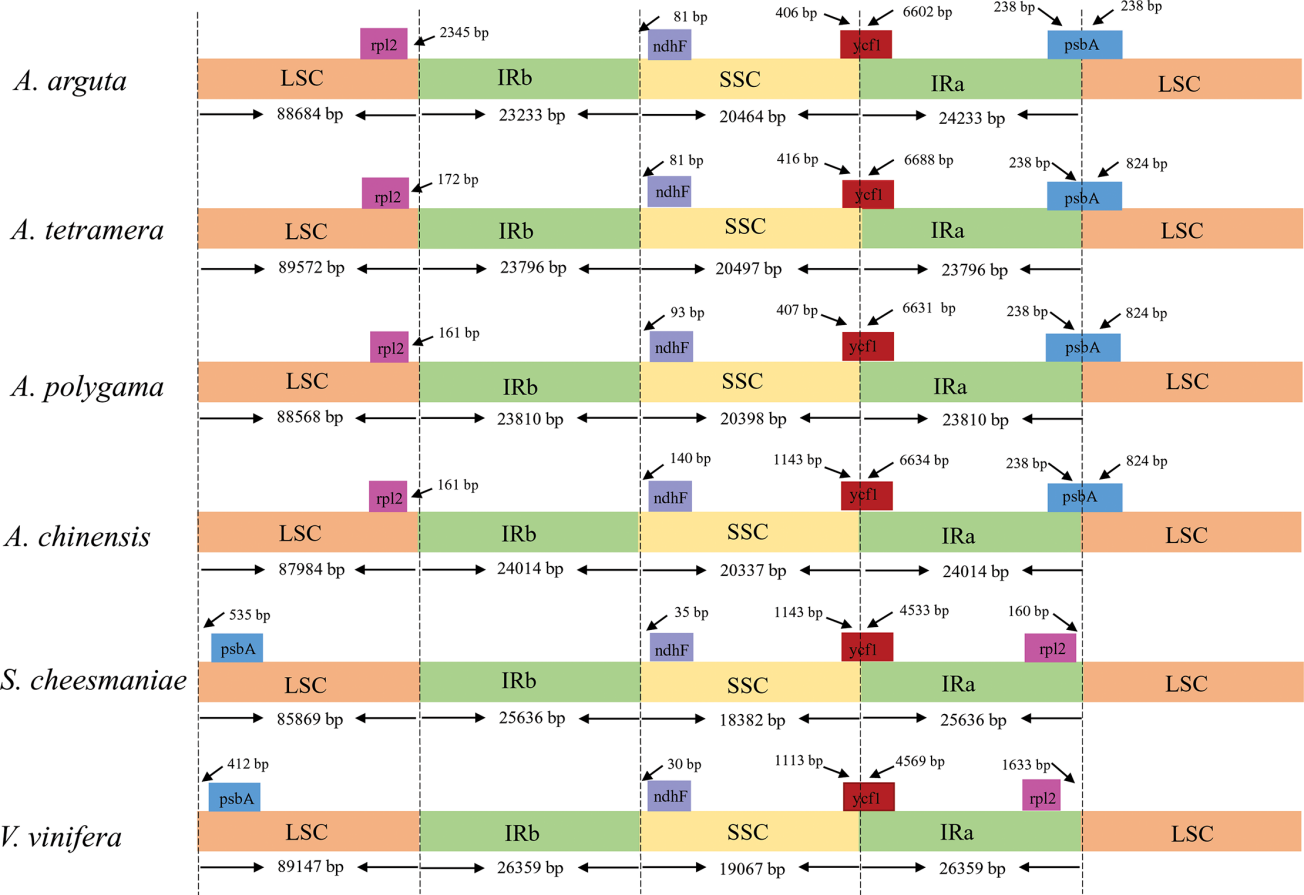
Comparison of the junction positions between the SSC and IR regions.

Expansion and contraction at the borders of the IR regions are common evolutionary events that often result in genome size variations in cp genomes[7,31]. Thus, the IR-LSC and IR-SSC borders in the *A*. *arguta* cp genome were compared with those of other *Actinidia*, including *A*. *polygama*, *A*. *chinensis*, and *A*. *tetramera*[8]as well as the closely related species *Vitis vinifera*[32] and *Solanum cheesmaniae*[33](Fig 7). The expansion of the *ycf1* gene influences the length of the cp genome by causing variability. The length of *ycf1* is 7,008 bp, 7,035, 7,038 bp, and 7,104 bp in *A*. *arguta*, *A*. *chinensis*, *A*. *polygama*, and *A*. *tetramera*, respectively, which is a little shorter than in *S*. *cheesmaniae* (5,686 bp) and in *V*. *vinifera* (5,686 bp). The *ndhF* genes from these 6 species are all located in the SSC, but the distance from the IRb boundary differs for each species. The *psbA* gene in *Actinidia* spans the IRa/LSC boundary, whereas this gene lies within the LSC in *S*. *cheesmaniae* and *V*. *vinifera*. Moreover, the *psbA* gene from *A*. *arguta* contracted in *Actinidia*. The *rpl2* gene is located in the LSC in all *Actinidia* but is located in the IRb region in the other sampled angiosperms. The variations at the IR/SC boundary regions in *Actinidia* lead to variations in their lengths and the lengths of their complete cp genome sequences.

### Phylogenetic analysis

**Fig 8:**
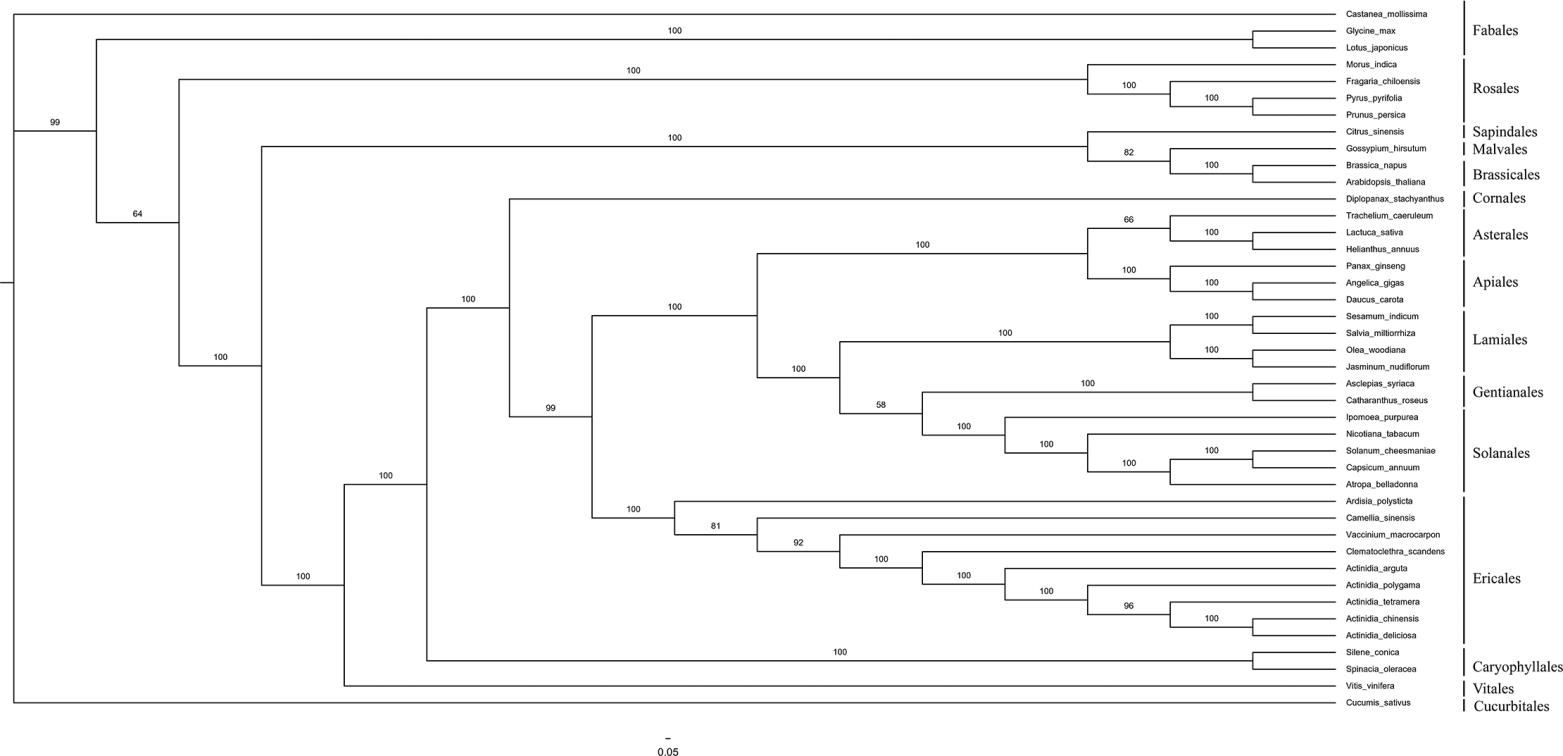
Phylogenetic tree reconstructed from the complete chloroplast genome sequences from forty-one species. Numbers above the lines represent the ML bootstrap values.

Sequences from the cp genome have been successfully used for phylogenetic studies in angiosperms[34]. *Actinidia* evolutionary relationships have been reported in previously published studies. Only a few *Actinidia* species have been evaluated by their complete cp genomes, though some have been studied using one or more cp loci[8,9,35]. In the present study, complete cp genomes, including our sequenced cp genome and forty-one other publicly available cp genomes, were used to perform phylogenetic analysis. The tree topology from the ML analysis is shown in Fig 8. The branching order among the major lineages of Eurosids, Euasterids I (Lamiids), Euasterids II (Campanulids), and basal asterids were consistent with those from a recent study[36]. A phylogenetic study of *Morella rubra* proved that complete cp genomes should be more regularly used to study relationships among angiosperms and to resolve the phylogenetic positions of various questionable lineages[7]. *A*. *arguta* was basal within *Actinidia*, and *Actinidia* was sister to *Clematoclethra* in basal asterids. The order of divergence in *Actinidia* was *A*. *polygama*, *A*. *tetramera*, *A*. *chinensis*, and *A*. *chinensis* var. *deliciosa*; the bootstrap values were 100%, except in the polygama clade, where the bootstrap support was 96%. We found that the phylogenetic relationship between *A*. *polygama* and *A*. *tetramera* based on the complete cp genome was incongruent with the relationship based on 56 common plastid protein coding genes in a previous report[8]. The genus *Actinidia* has a relatively complex phylogeny; Liu found that clades differed between gene trees and a species phylogeny[13]. The sister relationship between *A*. *chinensis* and *A*. *chinensis* var. *deliciosa* was consistent with the results from previous studies[37]. Phylogenetic studies using the complete cp genome are frequently used to study relationships among angiosperms, and their validity has been proven in many species[7,38].

### Conclusion

Cp DNA of *A*. *arguta* was extracted and the complete chloroplast genome sequence from *A*. *arguta* (157,611 bp) was obtained using the Illumina and PacBio RS II sequencing technologies. The structure and organization of this cp genome are very similar to those of the previously reported cp genomes in the genus *Actinidia*. The location and distribution of repeat sequences, SSRs, SNPs and indels were identified; sequence divergences in the LSC, SSC, and IR regions were also identified. Furthermore, ML phylogenetic trees were constructed based on the whole genome sequence. This study will be benefit investigations on evolutionary and population genetics in the *Actinidia* genus.

## Materials and methods

### Plant materials and cp genome DNA extraction

Leaves were selected from *A*. *arguta cv*.‘Ruby-3’ planted at Zheng Zhou Fruit Research Institute, CAAS, Zhengzhou, Henan, China (113° 71' E, 34° 71' N). High-quality cps were obtained by extraction and purification. The following are the steps for cp extraction: (1) leaves were ground with liquid nitrogen and then GR buffer (0.35 MD-sorbitol, 50 mM HEPES-KOH [pH 8.0], 2 mM EDTA-Na_2_ [pH 8.0], 1 mM MnCl_2_, 0.1% BSA, and 0.01% mercaptoethanol) was added at a W:V = 1:10 at 4°C and mixed for 20 min; (2) the mixed liquor was filtered two times with gauze, the filtrate was centrifuged (200 ×g, 4°C, 2 min), and then the precipitate was discarded and the supernatant retained; and (3) the solution was centrifuged at 2600 ×g at 4°C for 10 min and then the precipitate was resuspended in 5 ml GR to obtain the crude extract. The following at the steps for cp purification: (1) 5 ml crude extract, 10 ml 10% Percoll, 10 ml 32% Percoll and 10 ml 80% Percoll were added into a centrifuge tube and centrifuged at 8000 ×g for 35 min; (2) the layer with the green band was extracted after centrifuging and washed with washing buffer (0.35 M D-sorbitol, 50 mM HEPES-KOH [pH 8.0], 2 mM EDTA-Na_2_ [pH 8.0], 1 mM MgCl_2_, and 1 mM MnCl_2_; W:V = 1:5) and recentrifuged at 2600 ×g for 5 min at 4°C, and the precipitate was retained; (3) the precipitate was resuspended in washing buffer; and (4) cp intactness was examined under a microscope. Then, the cp DNA was extracted and tested using a Nano Drop 2000 (Thermo Scientific, Wilmington, USA) and agarose gel electrophoresis, and the qualified DNA was used for library construction[39,40].

### Library construction, sequencing and genome assembly

Illumina MiSeq library construction: A Bioanalyzer 2100 was used to test the library size, qPCR was used to quantify the library, and the validated DNA library was sequenced on an Illumina MiSeq Sequencing System following the manufacturer’s standard workflow. PacBio library construction: The steps were based on the PacBio Sample Net-Shared Protocol. The PacBio raw reads (polymerase reads) were filtered by discarding shorter and low-quality polymerase reads and discarding the adaptor sequences.

BLASR software was used to compare the PacBio data with the reference *A*. *chinensis* cp genome (KP297243) data and to extract the correct sequences for comparison[41]. The Sprai program was used to correct errors that occurred between the compared sequences[42], and the Illumina MiSeq data were used to correct the PacBio sequences using the Pilon software program[43]. The complete cp genome sequences from *A*. *arguta* werethen assembled using Organelle_PBA[44].

### Gene annotation

The complete cp genome sequences were predicted with the Chloroplast Genome Annotation, Visualization, Analysis and GenBank Submission Tool (CpGAVAS) and Dual Organellar GenoMe Annotator (DOGMA). The genes were annotated and categorized by Organellar Genome DRAW, and a physical map was constructed using Organellar Genome DRAW[45].

### Analysis of cp genome sequences

The repetitive structures, repeat sizes and locations of forward match (F), reverse match (R), palindromic match (P), and complementary match (C) nucleotide repeat sequences were identified by REPuter[45]. SSRs were detected using the MIcroSAtellite (MISA) identification tool with the default parameters[46].

Three complete cp genomes within the *Actinidia* genus, namely, *A*. *polygama*, *A*. *tetramera*, and *A*. *chinensis*, were selected for comparison with the genome of *A*. *arguta* (S1 Table). Collinearity was analyzed using the MUMmer software program[47] with the following filter parameters: (1) identity ≥80% and (2) length of collinearity regions ≥2000 bp. The sequence divergences between *A*. *arguta* and the three other *Actinidia* species were compared and plotted using mVISTA; *A*. *chinensis* served as a reference[48]. MUSCLE was then used to calculate the length and number of SNPs and indels[49].

### Phylogenetic analysis

Forty-one complete cp genomes representing 15 major lineages of angiosperms were included in the phylogenetic analysis (S1 Table), which was performed using the maximum likelihood (ML) method. One thousand replications were used to calculate the local bootstrap probability of each branch. RAxML was then used to construct the phylogenetic tree[50].

## Supporting information

S1 Table GenBank accession numbers used in the construction of the phylogenetic tree. (XLSX)

S2 Table Repeat structure distribution of *A*. *arguta*. (XLSX)

S3 Table Analysis of SNPs between *A*. *arguta* and other *Actinidia* species. (XLSX)

S4 Table Analysis of indels between *A*. *arguta* and other *Actinidia* species. (XLSX)
